# Voluntary exercise inhibits intestinal tumorigenesis in *Apc*^Min/+ ^mice and azoxymethane/dextran sulfate sodium-treated mice

**DOI:** 10.1186/1471-2407-8-316

**Published:** 2008-11-02

**Authors:** Jihyeung Ju, Bonnie Nolan, Michelle Cheh, Mousumi Bose, Yong Lin, George C Wagner, Chung S Yang

**Affiliations:** 1 Susan Lehman Cullman Laboratory for Cancer Research, Department of Chemical Biology, Ernest Mario School of Pharmacy, Rutgers, The State University of New Jersey, Piscataway, New Jersey 08854, USA; 2 Department of Neuroscience, Rutgers, The State University of New Jersey, Piscataway, New Jersey 08854, USA; 3 Department of Psychology, Rutgers, The State University of New Jersey, Piscataway, New Jersey 08854, USA; 4 Cancer Institute of New Jersey, New Brunswick, New Jersey 08903, USA

## Abstract

**Background:**

Epidemiological studies suggest that physical activity reduces the risk of colon cancer in humans. Results from animal studies, however, are inconclusive. The present study investigated the effects of voluntary exercise on intestinal tumor formation in two different animal models, *Apc*^Min/+ ^mice and azoxymethane (AOM)/dextran sulfate sodium (DSS)-treated mice.

**Methods:**

In Experiments 1 and 2, five-week old female *Apc*^Min/+ ^mice were either housed in regular cages or cages equipped with a running wheel for 6 weeks (for mice maintained on the AIN93G diet; Experiment 1) or 9 weeks (for mice on a high-fat diet; Experiment 2). In Experiment 3, male CF-1 mice at 6 weeks of age were given a dose of AOM (10 mg/kg body weight, i.p.) and, 12 days later, 1.5% DSS in drinking fluid for 1 week. The mice were then maintained on a high-fat diet and housed in regular cages or cages equipped with a running wheel for 16 weeks.

**Results:**

In the *Apc*^Min/+ ^mice maintained on either the AIN93G or the high-fat diet, voluntary exercise decreased the number of small intestinal tumors. In the AOM/DSS-treated mice maintained on a high-fat diet, voluntary exercise also decreased the number of colon tumors. In *Apc*^Min/+ ^mice, voluntary exercise decreased the ratio of serum insulin like growth factor (IGF)-1 to IGF binding protein (BP)-3 levels. It also decreased prostaglandin E_2 _and nuclear β-catenin levels, but increased E-cadherin levels in the tumors.

**Conclusion:**

These results indicate hat voluntary exercise inhibited intestinal tumorigenesis in *Apc*^Min/+ ^mice and AOM/DSS-treated mice, and the inhibitory effect is associated with decreased IGF-1/IGFBP-3 ratio, aberrant β-catenin signaling, and arachidonic acid metabolism.

## Background

Colorectal cancer is the second most common form of cancer in incidence and mortality in developed countries [1]. While its etiology remains unknown, it is thought that both genetic and environmental factors play critical roles in causing colorectal cancer. Epidemiological studies consistently show that physical activity is associated with a reduced risk of colon cancer [2-5]. However, the protective effect of exercise against colon carcinogenesis has not been consistently demonstrated in animal models [6-9]. The inconsistent results observed in animal studies is likely due to the nature of the animal model as well as the type of exercise employed. For example, voluntary exercise was shown to inhibit 1,2-dimethylhydrazine- or azoxymethane-induced colon tumorigenesis in rats [6,7], whereas forced exercise was shown not to inhibit intestinal tumorigenesis in *Apc*^Min/+ ^mice [8]. Mehl *et al*., however, noted that intestinal tumor formation in *Apc*^Min/+ ^mice was inhibited by forced exercise but not by voluntary exercise [9]. Colbert *et al*. reported that voluntary exercise combined with food deprivation and weight loss resulted in a reduction in tumor formation in male *Apc*^Min/+^mice [10]. Therefore, additional studies are required to evaluate the potential protective effects of physical exercise against intestinal tumor formation.

*Apc*^Min/+ ^mice carry a dominant heterozygous nonsense mutation at codon 850 of the mouse homologue of the human tumor suppressor gene, *APC *(*Adenomatous polyposis coli*). This mutation is significantly implicated in both sporadic and inherited human colorectal carcinogenesis [12-14]. *Apc*^Min/+ ^mice, therefore, are recognized as a genetically relevant animal model mimicking human intestinal carcinogenesis and have been utilized extensively for various chemoprevention studies [15]. Azoxymethane (AOM)-induced and dextran sulfate sodium (DSS)-promoted colon tumorigenesis in mice is an inflammation-associated colon carcinogenesis model that has been used in chemoprevention studies [11]. The present study was designed to investigate whether voluntary exercise inhibits intestinal tumorigenesis in two different animal models, *Apc*^Min/+ ^mice and AOM/DSS-treated mice. To further elucidate the mechanisms of the inhibition of tumorigenesis, the effects of exercise on insulin-like growth factor (IGF)-1 and IGF binding protein (IGFBP)-3 ratio, aberrant β-catenin signaling, and arachidonic acid metabolism were also investigated in the *Apc*^Min/+ ^mice.

## Methods

### Breeding and Genotyping of *Apc*^Min/+ ^Mice

Male *Apc*^Min/+ ^(C57BL/6J) and female wild-type littermate mice were initially purchased from The Jackson Laboratory (Bar Harbor, ME) as founders, and our own breeding colony was established in the animal facility of the Susan Lehman Cullman Laboratory for Cancer Research (Rutgers, The State University of New Jersey, Piscataway, NJ). Pups produced from the colony were weaned at 3 weeks of age. Genotyping was performed by routine PCR assays on tail DNA following the procedure previously described [16]. Five-week old female *Apc*^Min/+ ^mice were transferred to the animal facility of the Department of Psychology (Rutgers, The State University of New Jersey, Piscataway, NJ) for the exercise studies.

### Animal Treatment and Tissue Harvesting

All animal experiments were performed under protocols no. 02-027 and 87-060 approved by the Institutional Animal Care and Use Committee at Rutgers University. In Experiment 1, 5-week old female *Apc*^Min/+ ^mice on the AIN-93G diet (containing 7% fat as soy been oil, Research Diets, Inc.) were housed in regular cages or cages equipped with a running wheel for 6 weeks until the experiment was terminated at 11 weeks of age. In Experiment 2, 5-week old female *Apc*^Min/+ ^mice were placed on a high-fat diet (using AIN76A diet as the base by Dyets, Inc., Bethlehem, PA) containing 20% fat [17] and housed in regular cages or cages equipped with a running wheel for 9 weeks until the experiment was terminated at 14 weeks of age. The running wheel used was 7.0 cm in width and 16.5 cm in diameter for an inner circumference of 51.8 cm. Five mice were group-housed in each cage equipped with free access to the running wheel. Revolutions per day were measured by a digital counter (Fisher Scientific, Pittsburgh, PA) attached outside the running wheel. The total number of revolutions per day was converted to total kilometers per day. Mice were routinely checked for any abnormalities. All mice were euthanized by CO_2 _asphyxiation. Blood was taken by cardiac puncture (Experiment 2), allowed to clot for 2 h at room temperature, and then centrifuged for 30 min at 3,000 × *g*. The supernatant was retained as serum sample and frozen at -80°C. The small intestine and colon were removed, washed with ice-cold PBS, opened longitudinally, and flattened on filter paper. The number, location, and size of visible tumors in the entire intestine were determined under an illuminated magnifier (2×). Alternatively, the flattened tissues on filter paper were placed on dry ice briefly in order to score the visible tumors as described previously [16]. Visible tumors were then excised using microdissection scissors (Fine Science Tools. Inc., Foster City, CA) and frozen at -80°C.

In Experiment 3, male CF-1 mice at 6 weeks of age (Charles River Laboratories, Wilmington, MA) were given a dose of AOM (10 mg/kg body weight, i.p.) or the vehicle (sterile saline). Twelve days later, they were given 1.5% DSS (molecular weight of 36,000–50,000, ICN Biochemicals Inc., Costa Mesa, CA) in drinking fluid for 1 week. The mice were then placed on a high-fat diet (using AIN93M diet as the base by Research Diets, Inc., New Brunswick, NJ) containing 20% fat (mixture of 27% corn oil, 16% beef tallow, 10% lard, 12% butter fat, 30% hydrogenated soybean oil, and 5% peanut oil) [17] and housed in regular cages or cages equipped with a running wheel for 16 weeks. Five mice were group-housed in each cage equipped with free access to the running wheel. Body weights, food and fluid consumption, and general health status were monitored weekly. At the end of experiment, mice were sacrificed by CO_2 _asphyxiation. At necropsy, colons were removed, washed with ice-cold saline, opened longitudinally, flattened on filter paper, and fixed in 10% buffered formalin for 24 h. Visible colon tumors were scored in the fixed tissues. The length, width, and height of each tumor were measured using calipers (VWR International, INC., Bridgeport, NJ).

### Western Blot Analyses

Nuclear and postnuclear fractions (fraction without nuclear fraction) were prepared from frozen tumors using NE-PER Nuclear and Cytoplasmic Extraction Reagent Kit (Pierce Biotechnology, Lockford, IL) [16]. Protease and phosphatase inhibitor cocktails (Sigma) as well as *N*-acetyl-Leu-Leu-norleucinal (a proteosome inhibitor; Calbiochem, San Diego, CA) were added to extraction buffers from the kit. The protein concentration was determined using a bicinchoninic acid protein assay kit (Pierce Biotechnology). Tumor extracts (denatured at 95°C for 5 min in Laemmli sample buffer) containing equal amounts of protein were subjected to SDS-PAGE (4–15% gradient, Bio-Rad, Hercules, CA). Gels were transferred onto polyvinylidene difluoride membranes (Bio-Rad), and then the membranes were incubated with blocking buffer (Li-Cor Biosciences, Lincoln, NE) for 1 h at room temperature. Membranes were probed with primary antibody in blocking buffer at 4°C overnight. After washing with TBS containing 0.1% Tween-20 three times, the membranes were incubated with secondary antibodies conjugated to IR fluorophore, Alexa Fluor 680 (Molecular Probes, Eugene, OR) or IRDye 800 (Rockland Immnunochemicals, Gilbertsville, PA). Fluorescence was detected with the Odyssey Infrared Imaging System (Li-Cor Biosciences). β-Catenin and E-cadherin antibodies were purchased from BD Bioscience (San Jose, CA). β-Actin and histone H3 antibodies were purchased from Sigma and Cell Signaling Technology, Inc. (Beverly, MA), respectively.

### Enzyme Immunoassay

Serum IGF-1 and IGFBP-3 levels were determined using Quantikine Mouse IGF-1 Immunoassay kit (R&D System, Inc., Minneapolis, MN) and DuoSet Mouse IGFBP-3 Enzyme Immunoassay Development Kit (R&D System, Inc.) following the manufacturer's protocol. Molar ratio of IGF-1 to IGFBP-3 was calculated by 3.7 × IGF-1 (ng/ml)/IGFBP-3 (ng/ml) due to 1 ng/mL IGF-I = 0.130 n*M *IGF-I and 1 ng/mL IGFBP3 = 0.036 n*M *IGFBP-3.

Procedures for the Prostaglandin E_2 _(PGE2) enzyme immunoassay (EIA) were previously described [16]. In brief, the frozen tumors were placed into ice-cold T-PER tissue protein extraction buffer (Pierce Biotechnology) containing protease inhibitor cocktails (Sigma) and indomethacin (a cyclooxygenase inhibitor, Cayman Chemical, Ann Arbor, MI), and homogenized with 50 strokes in an ice-cold Dounce homogenizer (Wheaton, Millville, NJ). After centrifugation for 10 min at 12,000 × *g*, the supernatants were retained as a whole-tissue extract. Tissue extracts were acidified with HCl to pH 2.5, vortexed for 1 min after adding 800 μL of ethyl acetate, and then centrifuged at 3,000 × *g *for 10 min (Sorvall RT 6000B). The organic layer was collected and then dried under speed vacuum evaporator (VWR International, INC.). The dried samples were stored at -80°C until analysis. Each sample was reconstituted in EIA buffer (Cayman Chemical). Levels of PGE2 were measured using an EIA kit (Cayman Chemical).

### Statistical Analyses

For simple comparisons between two groups, two-tailed Student's *t-*tests were used. Since our prediction was that exercise would reduce tumor formation, the one-tailed Student's *t*-test was used as a back-up only if the two-tailed *t*-test showed borderline significance. Two-way analysis of variance (ANOVA) was used for combining data from more than one experiment (with two factors, *treatment *and *experiment*). Several normality tests (Anderson-Darling, Cramer-von Mises, Kolmogorov-Smirnov, and Shapiro-Wilk tests) were used for determining if a transformation of the response variable (tumor number) was needed.

## Results

### Effects of voluntary exercise on intestinal tumorigenesis and body fat levels in *Apc*^Min/+ ^mice on AIN93G or high-fat diets (Experiments 1 and 2)

In Experiment 1, female *Apc*^Min/+ ^mice maintained on the AIN93G diet in cages equipped with a running wheel for 6 weeks starting at 5 weeks of age ran approximately 2.8 km/mouse/day, with an assumption that only one mouse was on the running wheel at a time. As shown in Table 1, control female *Apc*^Min/+ ^mice that were housed in regular cages on the AIN93G diet had 29.2 ± 4.4 tumors in the small intestine and 0.6 ± 0.2 tumors in the colon. *Apc*^Min/+ ^mice that were housed in cages equipped with running wheels for 6 weeks had 31% fewer tumors in the small intestine than control *Apc*^Min/+ ^mice (p < 0.05). Only 0.4–0.6 colon tumors per mouse (<3% of the total intestinal tumors) were found. Neither the colon tumor multiplicity (Table 1) nor incidence (data not shown) was significantly affected by the voluntary exercise. The 6-week voluntary exercise resulted in a 51% decrease in omental fat pad weight/body weight (6.89 ± 1.02 mg/g versus 13.96 ± 1.57 mg/g in the control *Apc*^Min/+ ^mice; p < 0.05) and a 26% decrease in retroperitoneal fat weight/body weight (2.64 ± 0.37 mg/g versus 3.59 ± 0.43 mg/g in the control *Apc*^Min/+ ^mice). The voluntary exercise slightly increased food and fluid intake (~10%) without a significant influence on final body weights (18.9 g average). None of the groups exhibited noticeable signs of toxicity during the experimental period.

**Table 1:** Effect of voluntary exercise on intestinal tumor formation in *Apc*^Min/+ ^mice maintained on AIN93G diet or a high-fat^a^

**Group (N)**	**Small intestinal tumors**							**Colon tumors**
	**Region**			**Size**			**Total**	
		
	**Proximal**	**Middle**	**Distal**	**≤1 mm**	**1–2 mm**	**≥ 2 mm**		

*Experiment 1 on AIN93G diet (5 – 11 wk of age)*								
Control (27)	7.9 ± 1.4	-	21.3 ± 3.3	-	-	-	29.2 ± 4.4	0.6 ± 0.2
Exercise (28)	4.5 ± 0.9^c^	-	15.7 ± 3.0	-	-	-	20.2 ± 3.8^b^	0.4 ± 0.1
*Experiment 2 on High-fat diet (5 – 14 wk of age)*								
Control (19)	4.0 ± 0.4	12.1 ± 1.7	14.7 ± 2.0	18.0 ± 2.4	8.1 ± 1.3	4.8 ± 0.6	30.8 ± 3.4	0.6 ± 0.2
Exercise (19)	2.7 ± 0.3^c^	7.3 ± 1.6^c^	12.0 ± 1.4	15.3 ± 1.9	4.4 ± 0.9^c^	2.3 ± 0.4 ^d^	22.0 ± 2.8^c^	0.4 ± 0.1

^a ^Results of Experiments 1 and 2. In Experiment 1, the small intestine was divided into two segments (proximal and distal), and the tumor size was not scored because the majority of tumors in 11-week-old mice were ~1 mm in diameter. The number of mice was accumulated from 4 identical experiments that were done with smaller numbers of mice per group. Data were then combined and analyzed through a statistical adjustment; the effects of two factors, *treatment *(exercise) and *experiment*, as well as the interaction of *treatment *and *experiment*, on the response variable, *total small intestinal tumor numbers *(square-rooted to stabilize the variance), were initially assessed by two-way ANOVA; factors found not to affect the response variable significantly were excluded in the final statistical analyses. ^b^A statistically significant *treatment *(exercise) effect was found (p < 0.05). In Experiment 2, the small intestine was divided into three segments (proximal, middle and distal), and the tumor size was scored Each value represents mean ± SE of number of mice (N). ^c^: p ≤ 0.05 by two-tailed t-test;^d^: p < 0.005 by two-tailed t-test.

In Experiment 2, female *Apc*^Min/+ ^mice maintained on a high-fat diet in cages equipped with a running wheel for 9 weeks ran approximately 3.5 km/mouse/day. The 9-weeks of voluntary exercise resulted in a 29% decrease in tumor numbers in the small intestine (p = 0.05; Table 1), a value similar to the inhibition (31%) found in *Apc*^Min/+ ^mice on the AIN93G diet (Experiment 1 in Table 1). The voluntary exercise resulted in a more prominent decrease in the number of large size tumors (52% decrease in >2 mm tumors; p < 0.005) than the number of small size tumors (15% decrease in <1 mm tumors) in the small intestine. The 9-weeks of voluntary exercise resulted in a 34% decrease in omental fat pad weight/body weight (11.1 ± 5.9 mg/g versus 16.9 ± 1.8 mg/g in the control *Apc*^Min/+ ^mice; p < 0.02) and a 40% decrease in retroperitoneal fat weight/body weight (4.0 ± 0.6 mg/g versus 6.7 ± 0.8 mg/g in the control *Apc*^Min/+ ^mice; p < 0.01) without a significant influence on final body weights (20.8 ± 0.3 mg/g versus 21.3 ± 0.4 g in the control *Apc*^Min/+ ^mice). The exercise slightly increased food and fluid intake (~10%).

### Effect of voluntary exercise on serum IGF-1 and IGFBP-3 levels

In the female *Apc*^Min/+ ^mice maintained on the high-fat diet (Experiment 2), the 9-week long voluntary exercise increased serum IGFBP-3 levels by 19% (p < 0.03) and slightly decreased serum IGF-1 levels (by 9%), resulting in a 26% decrease in the molar ratio of IGF-1 to IGFBP3 levels (Table 3; p < 0.005).

**Table 3:** Effect of voluntary exercise on small intestinal tumor or serum levels of PGE2 in *Apc*^Min/+ ^mice^a^

Group (N)	PGE2 levels (% decrease)	
Experiment 1		
		
Small intestinal tumors (ng/mg protein)		
Control (11)	12.7 ± 1.4	
Exercise (10)	8.8 ± 0.8^b^	(31%)
		
Experiment 2		
		
Serum (pg/ml)		
Control (19)	436.4 ± 103.6	
Exercise (19)	390.3 ± 111.7	(11%)
		
Small intestinal tumors (ng/mg protein)		
Control (13)	26.5 ± 4.0	
Exercise (13)	20.5 ± 2.4	(23%)

^a^Samples from Experiments 1 and 2. PGE2 levels were determined in small intestinal tumor extracts and(or) serum samples by EIA. Values are mean ± SE of the number of samples (*N*) analyzed.

^b^p ≤ 0.05, two-tailed t-test.

### Effect of voluntary exercise on E-cadherin and nuclear β-catenin levels in small intestinal tumors

**Figure 1:**
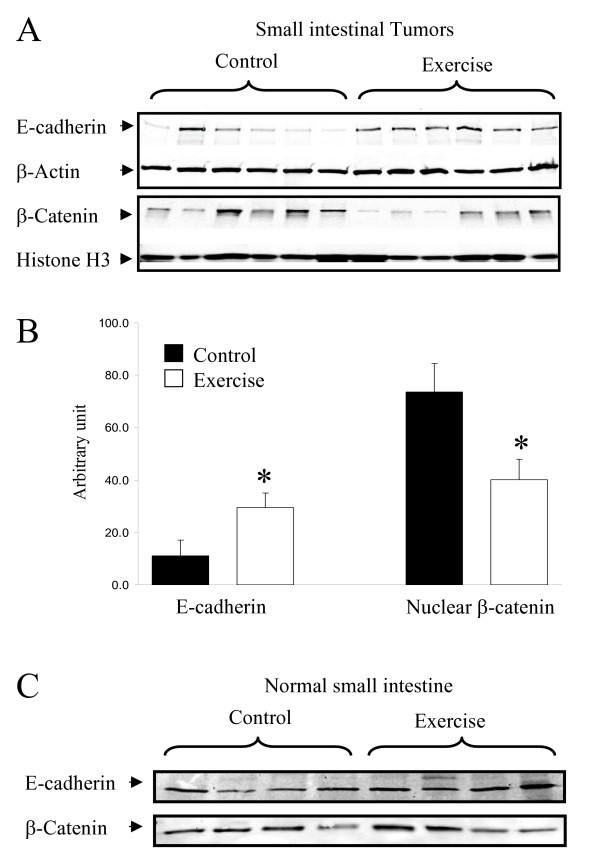
**Effects of voluntary exercise on protein levels of E-cadherin and nuclear β-catenin in small intestinal tumors and normal small intestine of *Apc*^Min/+ ^mice on AIN93G diet**. Samples from Experiment 1. **A**. Western blot analyses of postnuclear (for E-cadherin) and nuclear (for β-catenin) fraction of 6 representative tumor samples (out of a total of 9 samples analyzed) per group. Tumors used for these analyses were ~1 mm in the largest diameter. **B**. Protein levels were quantified by using Adobe Photoshop software (normalized by levels of β-actin for E-cadherin and levels of histone H3 for nuclear β-catenin). Columns: mean values (in arbitrary units) of the number of mice (9 per group); bars: SE; *: p < 0.03 by two-tailed t-test. **C**. Western blot analyses of postnuclear (for E-cadherin) and nuclear (for β-catenin) fraction of 4 representative normal small intestine samples per group.

By Western blot analyses, we measured levels of E-cadherin and nuclear β-catenin in small intestinal tumors of *Apc*^Min/+ ^mice maintained on the AIN93G diet (Experiment 1). As shown in Figures 1A and 1B, voluntary exercise resulted in a 2.6-fold increase in E-cadherin levels and a 46% decrease in the nuclear levels of β-catenin in the tumors (p < 0.03). In normal small intestine, exercise did not result in a significant change in those levels (Figure 1C).

### Effect of voluntary exercise on PGE2 levels in small intestinal tumors and serum

In the *Apc*^Min/+ ^mice maintained on AIN93G diet (Experiment 1), the 6-week voluntary exercise resulted in significantly decreased small intestinal tumor levels of PGE2 by 31% (Table 3; p < 0.05). In the *Apc*^Min/+ ^mice maintained on the high-fat diet (Experiment 2), the 9-week voluntary exercise resulted in decreases in serum levels of PGE2 by 11% (p = 0.05), respectively. In the small intestinal tumors, PGE2 levels were found to be decreased (by 23%) in the exercise group compared to those levels in the control group.

### Effects of voluntary exercise on colon tumorigenesis and body fat levels in AOM/DSS-treated mice on a high-fat diet (Experiment 3)

Male AOM/DSS-treated mice maintained on the high-fat diet in cages equipped with a running wheel for 16 weeks ran approximately 3.3 km/mouse/day. The 16-weeks of voluntary exercise resulted in a significantly lower number of colon tumors per tumor bearing mouse (49% of control, p < 0.05; Table 4). Voluntary exercise resulted in a 62% decrease in omental fat pad weight/body weight (p < 0.05) and a 60% decrease in retroperitoneal fat weight/body weight (p < 0.05). The voluntary exercise increased food and fluid intake (11% and 32%, respectively) but decreased final body weights (by 14%). The mice housed in cages equipped with a running wheel appeared to be more aggressive than the control mice in the regular cage. We observed that the mice in the exercise group often fought each other; most of these mice had scratch marks on their skin.

**Table 4:** Effect of voluntary exercise on body weights, regional fat weights, and colon tumor formation in AOM/DSS-treated mice on high-fat diet^a^

Groups (N)	Final body weight per mouse (g)	Omental fat weight/final body weight per mouse (mg/g)	Retroperitoneal fat weight/body weight per mouse (mg/g)	Tumor incidence	No. of tumor per mouse	No. of tumor per tumor-bearing/mouse	Average tumor dimension^b ^per tumor (mm)
Control (22)	49.9 ± 1.7	32.7 ± 3.6	10.6 ± 1.2	10/22 (46%)	1.9 ± 0.6	4.1 ± 1.0	2.7 ± 0.03
Exercise (17)	43.0 ± 1.2*	12.4 ± 2.6**	4.2 ± 1.0**	9/17 (53%)	1.1 ± 0.3	2.0 ± 0.2*	2.9 ± 0.07

^a^Results of Experiment 3. The mice were maintained on a high-fat diet and housed in regular cages or cages equipped with a running wheel for 16 weeks. Values are mean ± SE of the number of samples (*N*) analyzed.

^b^Average of length, width, and height of each tumor. There was no difference in the average volume of tumors (mm^3^) calculated using the formula *V *= 4/3π*r*^3 ^(*r *:the average dimention/2) between the two groups.

**p < 0.05 by two-tailed t-test. *p = 0.06 by two-tailed t-test and p = 0.03 by one-tailed t-test. Values with no superscript did not differ from the values of the control group statistically (p > 0.05)

## Discussion

The present study indicates that the opportunity to engage in voluntary running wheel exercise decreases the number of small intestinal tumors in female *Apc*^Min/+ ^mice maintained on AIN93G or a high fat diet by ~30% (Table 1) and the number of colon tumors (per tumor bearing mice) in male AOM/DSS-treated mice maintained on a high-fat diet by 51% (Table 4). Effects of voluntary or forced exercise on colon tumor formation in AOM/DSS-treated mice have not been reported, and the present study is the first that shows the inhibitory effect of voluntary exercise on colon tumorigenesis in this model.

Voluntary exercise has been shown previously to inhibit chemically-induced colon tumorigenesis in rats [6,7], but this beneficial effect has not been shown previously in the *Apc*^Min/+ ^mouse model [9]. Forced exercise was shown to inhibit tumorigenesis in male *Apc*^Min/+ ^mice in one study [9], but was without beneficial effect in a second study [8]. In addition, in previous studies, neither forced nor voluntary exercise were found to inhibit tumorigeneisis in female *Apc*^Min/+ ^mice [8,9]. The reasons for the inconsistent results from the different studies in *Apc*^Min/+^mice are not clear. One possible reason is the large variation in tumor yield among individual *Apc*^Min/+ ^mice (high variability across mice is often observed in our laboratory as well as in other research groups), which by chance might have resulted in false-negative results when small numbers of animals are used. Colbert *et al*. reported that voluntary exercise combined with food deprivation and weight loss resulted in a reduction in tumor formation in male *Apc*^Min/+ ^mice [10]. The authors concluded that the negative energy balance caused by the combination of food deprivation and exercise was the underlying mechanism for tumor reduction. In the present study, we found that voluntary exercise significantly inhibited small intestinal tumorigenesis in female *Apc*^Min/+ ^mice on a normal AIN93G or a high-fat diet as well as colon tumorigenesis AOM/DSS-treated male mice on a high-fat diet. Since mice in the present study were not food deprived, we conclude instead that exercise caused the inhibition of tumorigenesis under normal physiological conditions.

It was recently reported that voluntary running wheel exercise decreased the thickness of the dermal fat layer and inhibited ultraviolet B light-induced skin carcinogenesis in SKH-1 mice [18]. Also, a highly significant correlation between dermal fat thickness away from tumors and the skin tumor multiplicity was found across individual mice [18,19], suggesting that the exercise-induced reduction in dermal fat contributed to the inhibition of skin tumorigenesis. In order to assess the relationship between regional body fat and the number of intestinal tumors in individual mice in our study, we performed linear regression analysis. However, a statistically significant association between the weight of regional body fat (omental or retroperitoneal) and the number of intestinal tumors (in different sizes and locations) per mouse was not found (data not shown). In our study, *Apc*^Min/+ ^mice maintained on the high-fat diet had greater omental and retroperitoneal body fat weight/body weight (by 21% and 87%) than the *Apc*^Min/+ ^mice on AIN93G diet, but intestinal tumor numbers were not significantly different. Moreover, we previously reported that administration of caffeine decreased the two regional body fat weights, but did not inhibit intestinal tumorigenesis in the *Apc*^Min/+ ^mouse model [16]. Taken together, these results suggest that the reduction of body fat levels may not be responsible for the inhibition of intestinal tumorigenesis caused by voluntary exercise in the *Apc*^Min/+ ^mouse model.

High circulating levels of IGF-1 and low levels of IGFBP-3 (a major IGFBP that accounts for ~75% of IGF-1 bound) have been shown to be associated with an increased risk of several types of cancers, including colorectal cancer [20,21]. We found that voluntary exercise increased serum IGFBP-3 levels and decreased IGF-1/IGFBP-3 ratio (Table 2). The decrease in serum IGF-1 levels was not statistically significant (Table 2), and this is consistent with previous studies in the *Apc*^Min/+^mouse model [8,9] as well as other models [22,23]. In order to assess the relationship between IGFBP-3, IGF-1, or IGF-1/IGFBP-3 ratio and intestinal tumor numbers in individual mice, linear regression analysis was performed with data from 38 mice (in Experiment 2), but no significant associations were found (data not shown). It is still of interest, however, to further investigate how voluntary exercise increases IGFBP-3 levels and how this effect could contribute to the inhibitory action on intestinal tumorigenesis.

**Table 2:** Effect of voluntary exercise on serum IGF-1 and IGFBP-3 levels in *Apc*^Min/+ ^mice on high-fat diet^a^

Groups (N)	IGF-1 levels (ng/ml)	IGFBP-3 levels (ng/ml)	Molar ratio of IGF-1 to IGFBP-3^b^
Control (19)	312.4 ± 14.5	53.5 ± 2.8	22.8 ± 1.6
Exercise (19)	283.0 ± 18.8	63.9 ± 3.4*	16.8 ± 1.6**

^a ^Samples from Experiment 2. IGF-1 and IGFBP-3 levels were determined in serum samples by EIA. Values are mean ± SE of the number of samples (*N*) analyzed.

^b^Molar ratio was calculated by 3.7 × IGF-1 (ng/ml)/IGFBP-3 (ng/ml) due to 1 ng/mL IGF-I = 0.130 n*M *IGF-I and 1 ng/mL IGFBP3 = 0.036 n*M *IGFBP-3.

*p < 0.03;**p < 0.005; two-tailed t-test.

The observed increase of E-cadherin levels and decrease of nuclear β-catenin levels in tumors (Figure 1) may contribute to the inhibition of tumorigenesis in *Apc*^Min/+ ^mice by exercise because aberrant β-catenin signaling is a key molecular event in the development of intestinal tumors in this model [24]. Similar events have been associated with the cancer preventive activities of (-)-epigallocatechin-3-gallate (EGCG), the major polyphenolic compound found in green tea [16]. The observed decrease in levels of PGE2 in serum and tumors (Table 3) may also contribute to the inhibition of intestinal tumorigenesis. It has been well demonstrated that reduction of PGE2 production decreases colorectal carcinogenesis [25]. A high level of leisure-time physical activity has been reported to be associated with low PGE2 levels in rectal mucosa [26].

In the present study, five mice were group-housed in each cage; therefore, we could not determine how much each mouse ran each day. We did observe that 2–4 mice could run on the running wheel together. Nonetheless, it is possible that dominant mice gained more assess to the wheel than subordinates. However, if this were the case, we would predict high variability in the exercise group on measures of tumor number, body weight, fat weight, etc. In the present study, the variability observed in these parameters in the exercise groups was similar to that observed in the corresponding control groups. Therefore, although there were five mice per cage, we conclude they each contributed about equally to the number of wheel revolutions.

## Conclusion

We found that voluntary running wheel exercise inhibited small intestinal tumor formation in female *Apc*^Min/+ ^mice and colon tumor formation in AOM/DSS-treated male CF-1 mice, and the inhibitory effect may be associated with decreased IGF-1/IGFBP-3 ratio as well as aberrant β-catenin signaling and arachidonic acid metabolism. Further studies are needed to better characterize the mechanisms of cancer prevention by exercise.

## Competing interests

The authors declare that they have no competing interests.

## Authors' contributions

JJ participated in designing experiments, breeding & genotyping mice, and sacrificing animals, and performed tumor-scoring, tissue-harvesting, Western blotting, ELISA, data analyses, and manuscript writing & submission. BM&MC performed animal experiments and participated in sacrificing animals and data analyses. MB participated in breeding, genotyping, and sacrificing mice. YL performed two-way ANOVA and normality tests in statistical analyses. GCW co-conceived the study with CSY and helped from designing experiments to writing manuscript. CSY conceived the study, coordinated different experiments, and revised the manuscript. All authors read and approved the manuscript.

## Pre-publication history

The pre-publication history for this paper can be accessed here:


